# IC Regimen: Delaying Resistance to Lorlatinib in ALK Driven Cancers by Adding Repurposed Itraconazole and Cilostazol

**DOI:** 10.3390/cells13141175

**Published:** 2024-07-10

**Authors:** Richard E. Kast

**Affiliations:** IIAIGC Study Center, Burlington, VT 05408, USA; richarderickast@gmail.com

**Keywords:** ALK kinase, cilostazol, Hedgehog, itraconazole, large cell neuroendocrine cancer, non-small cell lung cancer, platelets

## Abstract

Lorlatinib is a pharmaceutical ALK kinase inhibitor used to treat ALK driven non-small cell lung cancers. This paper analyses the intersection of past published data on the physiological consequences of two unrelated drugs from general medical practice—itraconazole and cilostazol—with the pathophysiology of ALK positive non-small cell lung cancer. A conclusion from that data analysis is that adding itraconazole and cilostazol may make lorlatinib more effective. Itraconazole, although marketed worldwide as a generic antifungal drug, also inhibits Hedgehog signaling, Wnt signaling, hepatic CYP3A4, and the p-gp efflux pump. Cilostazol, marketed worldwide as a generic thrombosis preventative drug, acts by inhibiting phosphodiesterase 3, and, by so doing, lowers platelets’ adhesion, thereby partially depriving malignant cells of the many tumor trophic growth factors supplied by platelets. Itraconazole may enhance lorlatinib effectiveness by (i) reducing or stopping a Hedgehog-ALK amplifying feedback loop, by (ii) increasing lorlatinib’s brain levels by p-gp inhibition, and by (iii) inhibiting growth drive from Wnt signaling. Cilostazol, surprisingly, carries minimal bleeding risk, lower than that of aspirin. Risk/benefit assessment of the combination of metastatic ALK positive lung cancer being a low-survival disease with the predicted safety of itraconazole-cilostazol augmentation of lorlatinib favors a trial of this drug trio in ALK positive lung cancer.

## 1. Introduction

This paper presents the data analysis behind the IC Regimen, itraconazole, cilostazol augmentation of lorlatinib. That analysis of published data collected over the last 20 years on the biochemistry and physiological effects of two drugs from general medical practice, itraconazole and cilostazol, intersect with ALK kinase (ALK) non-small cell lung cancers’ pathology to contribute to lorlatinib’s growth suppression of that cancer.

Lorlatinib is an ALK inhibitor with good brain tissue penetration, effective in treating cancers having increased drive by ALK overexpression or overactivity [1,2,3]. About 5% of non-small cell lung adenocarcinomas (NSCLC) are ALK positive. Several other cancers that commonly have an aberrant ALK driven growth drive are large cell neuroendocrine lung cancer (LCNEC), glioblastoma, neuroblastoma, anaplastic large cell lymphoma, and anaplastic thyroid carcinoma. Cross-covering growth signaling pathways (bypass signaling mechanisms) such as that of EGFR, KIT, MET, BRAF, or others can compensate for lorlatinib-induced shut down of ALK [4,5].

Inhibition of lorlatinib’s main catabolic enzymes, CYP3A and UGT1A4, resulted in a 16 times elevation of mouse brain tissue levels, but that elevation was asymptomatic [6]. Lorlatinib has a wide therapeutic index. Common side effects of lorlatinib include edema, headache, hyperlipidemia, and neuro-psychiatric impairments. These are usually treated by standard means but occasionally require dose reduction [7,8,9].

Non-pathological ALK is activated by any one of several endogenous ligands binding to the ALK extracellular domain. Such binding triggers dimerization and autophosphorylation of the ALK intracellular domain that in turn triggers a downstream signaling chain. Pathological ALK can be activated this way too, but has other activations mechanisms as well, vide infra.

In general, a cancer’s resistance to pharmacological ALK inhibition occurs by one or more of these paths [1,4,5,10,11,12]: 

(i) via activation of bypass survival pathways, EGFR, or the insulin-like growth factor (IGF) for example, or

(ii) via further mutation of ALK, or

(iii) via ALK gene amplification, or

(iv) via simple compensatory increases in ALK translation, or

(v) via upregulation of cell lorlatinib efflux pumps.

Pharmacological inhibition of ALK itself will provoke a homeostatic upregulation of ALK protein expression [10].

In a quarter of cases, the origin of resistance to lorlatinib is unknown. But, in any case, resistance supervenes over time, leading to clinical relapse [12,13].

This paper recounts data showing the mechanisms by which two repurposed drugs from general medical practice—itraconazole and cilostazol—intersect with ALK signaling systems and lorlatinib action to potentially augment lorlatinib’s effect or undermine aspects of a cancer’s development of resistance to lorlatinib.

Table 1 and Table 2 list some of the most basic pharmacological parameters of the three IC Regimen drugs.

**First Preface:**

“All moves create weaknesses and strengths.” An aphorism in chess but applicable to oncology.

**Second Preface:**

“The decisive operation will directly achieve the purpose of the mission. All other operations that facilitate success of the decisive operation are shaping operations.” 

An aphorism in military doctrine but applicable to oncology.

**Third Preface:**

“He who tries to hold onto everything holds onto nothing.” King Fredrick the Great, 1712-1786, King, musician, composer, and military theorist

The three Prefaces above refer to several basic principles of metastatic cancer treatment that underlie development of the IC Regimen. These principles are enunciated more fully elsewhere [14,15]. The core conclusion from these principles, as encapsulated in the three aphorisms, is that until and unless a “silver bullet” is found, we require a multidrug approach to metastatic NSCLC, accepting the risks attendant to multidrug approaches.

## 2. ALK

ALK has an extracellular region, a single transmembrane helix and an intracellular tyrosine kinase domain [16,17]. When ALK oligomerizes in the absence of a ligand, retention in the cytosol results [18]. ALK oligomers or dimers activate the kinase domain, triggering downstream signaling of Ras/Raf/MEK/ERK1/2 and JAK/STAT pathways [19]. ALK is essential for embryological development but is not usually much expressed in adult tissue.

Side effects of slowed speech and other neurocognitive problems are common, as are elevated cholesterol and triglycerides. Side effects tend to resolve quickly after stopping or dose reduction [20]. Resistance develops often within the first few years of an initially responsive ALK positive NSCLC [21]. MYC transcription factor drives ALK expression, and ALK signaling drives MYC expression thus forming a potential mutually reinforcing amplification feedback loop, MET→ALK→MET→ALK→… [22,23]. Two other ALK related amplification loops are detailed below.

## 3. Hedgehog

Concomitantly upregulated Hedgehog signaling (Hh) in ALK positive cancers can complicate treatment with any ALK inhibitor [24]. In general, Hh signaling by itself can become a link in a cancer’s growth and survival signaling, engaged as part of the signaling chain initiated by other growth driving systems [25,26,27]. The end result of the Hh signaling complex is the creation of Gli transcription factors that bind to their consensus binding sites, either a Gli-R that represses or a Gli-A that activates the target genes’ transcription.

NPM-ALK fusion protein in lymphoma, for example, results in increased Hh signaling. NPM-ALK is a constitutively active fusion ALK in some lymphomas that enhances creation of transcription activating Gli-A [28].

**Figure 1:**
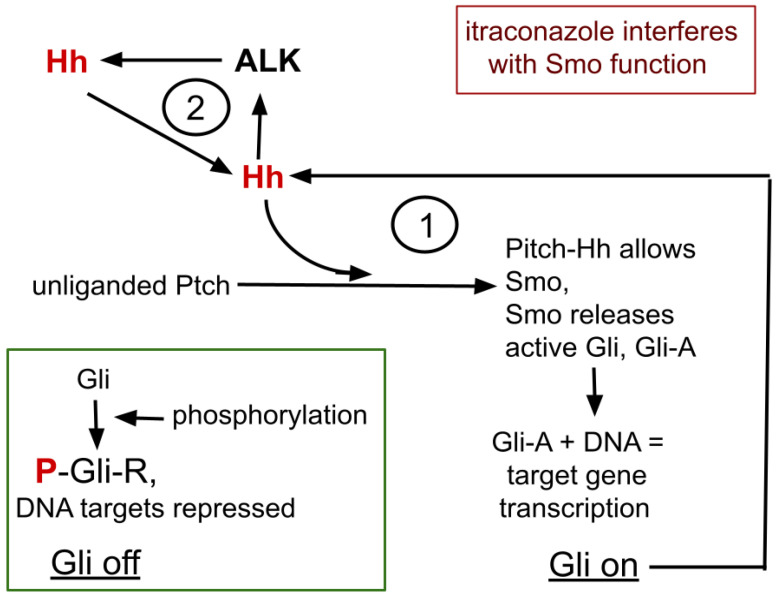
The core process of itraconazole’s interaction with ALK and Hh signaling. Omitted from this diagram are many intermediate steps, many cofactors that enhance or inhibit the isoforms of Gli. 1 refers to the bistable feedback cycle within Hh signaling. 2 indicates the positive feedback loop between ALK and Hh.

A simplified overview of the Hh signaling complex and its relationship with ALK expression is graphically presented in Figure 1 and recounted here. Four core proteins of Hh signaling, Gli, Hh, Ptch, and Smo, interact in Hh signaling. Gli is the central element that, by differential processing, either represses or promotes target gene transcription.

Ptch has an extracellular receptor domain and an intracellular effector domain. In the quiescent, unliganded Hh signaling complex, Ptch prevents Smo from access to Gli, allowing Gli’s sequential phosphorylation, protein kinase A (PKA)→glycogen synthase kinase 3-beta (GSK3)→casein kinase I, creating the Gli repressor form (Gli-R) that then binds to consensus DNA areas to repress expression and translation of the many Hh target genes, one of which is for Gli itself, forming the feedback cycle marked 1, in Figure 1.

Hh→Gli-A→Hh→Gli-A→…

Thus creating within Hh signaling and Gli, a bistable switching system with potential positive amplifying feedback loop within that system [29,30].

Thus Gli signaling tends to have two stable states,

(i) Gli-A as transcription promoter increases Gli transcription, and

(ii) Gli-R as transcription repressor represses Gli transcription.

After the Hh signaling complex binds an Hh ligand, Ptch is replaced by Smo, allowing release of Gli without undergoing the phosphorylation chain. Gli then becomes an active target DNA translation promoter, Gli-A.

Dozens of stimulating or inhibiting factors influence this simplified schematic, as do other post-translational Gli modifications. High GLI-A to GLI-R ratios are mainly associated with proliferation, increased survival, and stem cell self-renewal, while low ratios favor differentiation and quiescence [29,31,32].

## 4. ALK and Hh Form a Cyclic Amplifying System

Hh and ALK systems can interact. Hh is amplified in ALK positive lymphoma where silencing GLI inhibits growth of ALK driven lymphoma cells [33,34]. ALK inhibition suppresses functioning GLI-A transcription factor and active ALK signaling triggers increase in Gli-A, thus forming a second amplification feedback loop within the ALK-Hh system, as depicted as feedback cycle 2, in Figure 1 [28,33,35].

ALK→Hh→ALK→Hh→…

Hh signaling itself contributes to growth and neuroendocrine lineage selection in LNEC [36,37,38]. Ishiwata et al. showed that Gli inhibition or quantitative Gli reduction suppressed growth in an experimental model of LCNEC [39]. Hh is best recognized as a driver of basal cell carcinoma and medulloblastoma, but is seen in some cases of breast, lung, prostate, and other cancers as well.

So, we see two amplification feedback loops within the ALK/Hh system, shown in Figure 1. Hh functioning diminishment therefore has potential to allow lorlatinib to remain effective.

## 5. Hedgehog Signaling

### 5.1. Hh and the Repurposed Drug Itraconazole

In the antifungal role, itraconazole inhibits fungal lanosterol 14-α-demethylase, preventing ergosterol that is required for fungal wall formation. In the anti-cancer role, itraconazole inhibits Hh signaling by binding Smo [40,41,42,43,44].

A few other intersections of Hh with ALK:

1. Hh inhibition with vismodegib or itraconazole clinically suppresses, but often incompletely so, growth of basal cell carcinoma [40,45,46]. Approximately 85% of sporadic basal cell carcinoma carry mutations in Hh pathway genes, especially in PTCH, SUFU, and SMO genes, any of which tends to lead to the aberrant activation of GLI.

ALK and Hh are also related in basal cell carcinomas. Basal cell carcinoma generally has >250 fold increase in ALK and its ligands, pleiotrophin and midkine, compared to normal epidermis. Stronger expression of phosphorylated ALK in basal cell carcinoma tumor nests than normal skin was observed by immunohistochemistry [47].

2. A binding site for GLI resides in the *ABCG2* efflux pump’s consensus sequence promoter. Accordingly, Gli-A increases ABCG2 protein (synonymous with BCRP) expression, a stem cell marker in NSCLC [48,49,50].

### 5.2. Itraconazole Caveats

Itraconazole’s absorption is erratic. It requires an acidic environment for ideal absorption. Proton pump inhibitors must be avoided and itraconazole must be given with an acidic beverage like Coke™, pH2, or orange juice. It is difficult to draw conclusions from studies that did not assure these conditions.

Sun sensitivity occurs occasionally with itraconazole, so it is recommended to use an SPF 50 umbrella and to avoid direct sunlight.

Lorlatinib is metabolized by CYP3A4 and UGT1A4m. Itraconazole increases lorlatinib Cmax by 24% and its systemic exposure by that strong CYP3A3 inhibition [51,52]. The combination of lorlatinib plus itraconazole therefore requires monitoring for adverse events from increased lorlatinib levels. Human study of lorlatinib showed addition of itraconazole raised lorlatinib plasma Cmax by 124%, the AUC by 142% [53].

Lorlatinib cellular efflux is partially mediated by p-pg (P-glycoprotein, synonymous with MDR1 and ABCB1). Itraconazole also inhibits p-gp [54,55]. Together these two attributes raise risk of increased CNS side effects by virtue of both elevation of blood level through CYP3A4 inhibition and by lowering blood-brain barrier drug efflux by p-gp inhibition. The lorlatinib package insert from the manufacturer states “Avoid concomitant use of LORBRENA [lorlatinib] with a strong CYP3A inhibitor. If concomitant use cannot be avoided, reduce the LORBRENA dosage” [https://lorbrena.pfizerpro.com/].

In addition to Hh signaling, itraconazole inhibits an unusually wide range of human enzymes and signaling systems:

1. p-gp = MDR1 = ABCB1 [55]
2. CYP3A [56]
3. ABCG2 = BCRP [55,57].
4. Human 11β-hydroxysteroid dehydrogenase 2 [58]
5. Human Niemann-Pick C1 lysosomal protein [59,60]
6. Human mitochondrial protein voltage-dependent anion channel 1 (VDAC1) [59,60]
7. Human CEBPB, a transcription factor [61]
8. 5-lipoxygenase [62]
9. Wnt signaling [43,63,64,65,66]

However, the low incidence of side effects from itraconazole when used as an antifungal drug would indicate either (i) low degree of inhibition in practice or (ii) the existence of readily engaged cross-covering systems during clinical use. Either could lower clinical effectiveness during cancer treatment. Also, since many commonly used drugs are catabolized by CYP3A4, itraconazole has potential for drug-drug interactions that must be kept in mind when considering what other drugs might be given.

The main side effects of increased lorlatinib systemic exposure are worsening of hypertriglyceridemia, hypercholesterolemia, and psychiatric or cognitive disturbances. These are potentially treatable by standard means but may require lorlatinib dose reduction. Of note here is the potential of cilostazol to lower hypertriglyceridemia and hypercholesterolemia [8,9].

### 5.3. Additional Potential Benefits of Itraconazole

In addition to itraconazole’s actions as antifungal drug and Hh inhibition, several other itraconazole attributes and findings make it an attractive adjunct in treating NSCLC.

1. Itraconazole also inhibits Wnt signaling [43,63,64,65,66]. Overactive Wnt signaling system contributes to NSCLC growth vigor and treatment resistance [67,68,69,70].

2. Perhaps the most compelling data favoring the use of adjunctive itraconazole are clinical experiences we already have with itraconazole repurposed as adjuvant for NSCLC treatment with surgery or traditional cancer cytotoxic chemotherapy.

(i) NSCLC cases in platinum-based chemotherapy with itraconazole 200 mg/day, 21 days on, 7 days off, (rationale for days off itraconazole unknown) experienced longer progression free survival but the same 1 year survival rate as those in the same chemotherapy without itraconazole [71].

(ii) Adding itraconazole (600 mg/day) alone prior to surgery in NSCLC cases resulted in lung tumor size and perfusion reduction after 14 days of use. Tumor tissue levels of itraconazole exceeded those in plasma [72].

(iii) The CUSP9v3 trial, which included continuous itraconazole 200 mg twice daily over years in recurrent glioblastoma, showed evidence of benefit and good tolerance [15,73].

(iv) Itraconazole 600 mg/day lengthened the PSA doubling time in advanced prostate cancer without lowering androgen levels. Important to note here is that 200 mg itraconazole was without effect [74,75].

(v) In advanced NSCLC cases given pemetrexed with itraconazole 200 mg/day, 21 days on, 7 days off survived longer than those given pemetrexed alone, 32 months versus control 8 months [76].

(vi) Itraconazole had a rather dramatic effect in prolonging survival in women being treated for ovarian cancer [77,78]. Given these data are from 2014, it is unclear why these studies have not been verified or refuted, nor is it clear why itraconazole is not routinely used in treating ovarian cancers in 2024.

## 6. Repurposed Drug Cilostazol

This section reports data on the trophic function of platelets in cancer growth generally and in NSCLC specifically and how deprivation or reduction of platelets’ trophic function by cilostazol may retard NSCLC growth or delay lorlatinib resistance.

### 6.1. Cilostazol

Cilostazol is an oral drug marketed for treatment of intermittent claudication. It inhibits phosphodiesterase 3 (PDE3) and the adenosine uptake pump. PDE3 mediates the conversion of c-AMP to AMP.

In clinical use for 20+ years, cilostazol inhibits platelet aggregation and causes vasodilation when used in treating peripheral arterial and cerebrovascular disease [79,80]. Standard treatments are usually effective for cilostazol side effects of mild headache and diarrhea [81].

Cilostazol inhibits platelet aggregation induced by collagen, ADP, epinephrine, arachidonic acid, and other common aggregation or activation stimuli, reducing the number of activated platelets in circulation yet with minimal bleeding risk [82,83,84,85,86].

Given these attributes it is unclear why cilostazol does not carry more of a bleeding risk. About 1 per 100 patient-years of people with a previous stroke treated with cilostazol for secondary stroke prevention will experience a serious bleeding event [87]. Surprisingly, comparative anti-platelet studies show a lower bleeding risk with cilostazol than with low dose aspirin (81 mg/day) [88,89,90,91].

Cilostazol is metabolized by hepatic CYP3A4 and 2C19 with circulating half-life about 12 h. Simultaneous itraconazole use, therefore, has potential to increase cilostazol’s effects and halflife. Importantly for potential use in NSCLC, bleeding time is not prolonged by cilostazol but is prolonged by aspirin and ticlopidine even though all three show similar clinical thrombosis protection and similar ex vivo platelet aggregation inhibition [86,92].

The main physiological consequences of cilostazol are reduced vascular smooth muscle proliferation, reduced platelet activation, and reduced platelet aggregation [93,94,95].

PDE3 catalyzes the reaction of cAMP to AMP. PDE3 has high competitive affinity for both cAMP and cGMP but PDE3 does not catalyze a parallel reaction of cGMP to GMP [96].

The term “platelet activation” refers to a process that prepares or permits platelet degranulation of intracellular contents. Activation occurs with platelet exposure to ADP, thrombin, vitronectin, fibronectin, or thrombospondin-1, as examples. Multiple other factors can trigger platelet activation. Platelet alpha-granules contain dense concentrations of Factors V, IX, XIII, antithrombin, thrombospondin, CXCL1, CXCL4, CXCL5, IL-8, CCL2, MCP-1, CCL3, CCL5 (RANTES), epidermal growth factor (EGFR), hepatocyte growth factor (HGF), IGF, TGF-beta, vascular endothelial growth factor (VEGF), fibroblast growth factor (FGF), and platelet derived growth factor (PDGF) [97,98,99]. Notably, platelets contain fully 25% of blood borne VEGF [100,101,102]. By adhering to tumor stroma then releasing their contents, platelets contribute these growth factors to the growing tumor vasculature and to the malignant cells themselves [103].

Platelet borne alpha-granules are a rich source of HGF. HGF is the cognate ligand for c-MET. Development of c-MET amplification and the MET→ALK→MET→ALK→…

amplifying feedback loop constitutes one of the resistance mechanisms to lorlatinib [11,104]. Therefore, reduction of platelets provision of HGF by cilostazol potentially delays that resistance pathway.

### 6.2. Trophic Function of Platelets in Cancer

Platelets play important, multifaceted roles in cancer growth and metastasis establishment [105,106,107,108,109,110,111]. Elevated platelet count is a negative prognostic sign across the common cancers [103,112,113]. Specifically in pulmonary LCNEC [114,115] and NSCLC [116,117,118,119,120], a higher platelet-to-lymphocyte ratio (PLR) predicts shorter OS.

**Figure 2:**
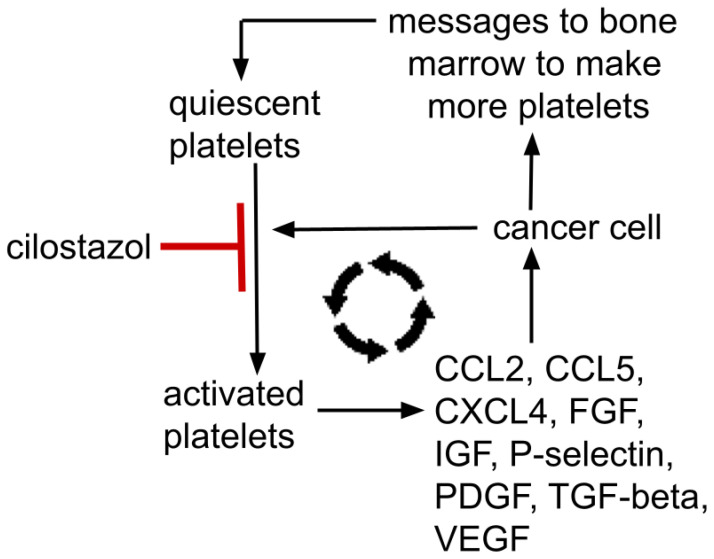
Mutually supporting feedback loop between a cancer and platelets; platelet→metastasis facilitation→tumor signals bone marrow to make more platelets→increased platelets→increased tumor mass→…etc. Platelet alpha-granules contain dense concentrations of Factors V, IX, XIII, antithrombin, thrombospondin, CXCL1, CXCL4, CXCL5, IL8, MCP-1, MIP-1α, CCL5, EGFR, HGF, IGF, TGF-beta, VEGF, FGF, PDGF. P-selectin resides on platelets’ surface. These growth factors are representative, not all-inclusive. Cilostazol’s platelet’s activation inhibition is moderate, not absolute.

**Figure 3:**
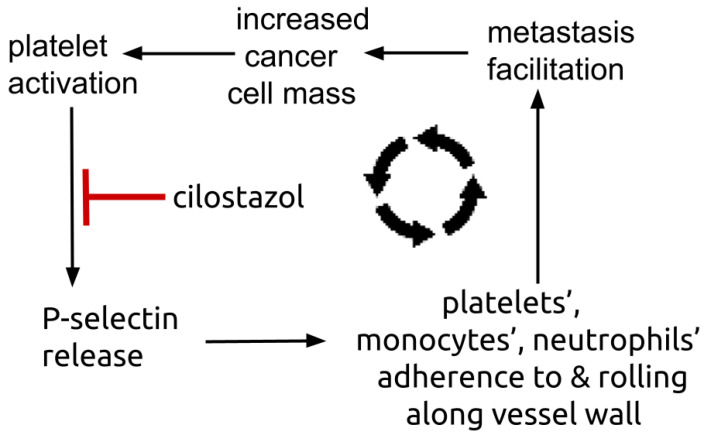
Diagram showing platelets’ P-selectin centered amplifying feedback loop. Platelets’ surface P-selectin enables platelets, monocytes, and neutrophils to adhere to and roll along vessel endothelium, a process that facilitates metastasis establishment. Metastasis establishment increases total tumor mass that in turn triggers further number of activated, trophic platelets.

Gouban et al. and others have reported the existence of tumor-induced platelet activation, otherwise termed “tumor educated platelets”. This results in a reciprocal relationship where tumors change, educate, and condition platelets that then contribute to tumor growth and dissemination, a relationship documented in several cancers [113,121,122,123]. See schematic representations of this in Figure 2 and Figure 3.

Thus, yet another positive feedback loop exists where platelets help a cancer grow and a growing cancer signals the bone marrow to make more platelets and educates those platelets to release growth enhancing trophic factors that further tumor growth, etc. This mutually supporting process is depicted in Figure 2. Such a reciprocal tumor-platelet mutually supporting system is recognized across common cancers.

Cilostazol inhibited ex vivo platelet-dependent fibrin formation and platelet release of CCL5 and CXCL4 [124].

CCL5 synthesized by NSCLC cells and by their stroma enhance NSCLC growth, metastasis, and trophic myeloid cell chemotaxis to tumor [125,126,127,128,129]. It has not been determined yet to what degree deprivation of platelets’ contribution of CCL5 will affect growth. Regarding CXCL4, NSCLC cases with higher CXCL4 levels had worse overall survival than did cases with lower expression [130].

By whole-blood flow cytometry, leukocyte-platelet aggregates mediated by platelets’ surface P-selectin depend on degranulation of alpha-granules [131]. Preliminary evidence implicates these leukocyte-platelet aggregates in facilitating metastases [132,133,134].

Cilostazol decreases both circulating and platelet released P-selectin [135,136,137]. Plasma P-selectin is elevated in NSCLC, where greater elevations predispose to vascular thrombosis events compared to those with lesser elevations [138]. Treatment with cilostazol 100 mg twice daily lowered blood platelet-neutrophil aggregates and plasma P-selectin in peripheral artery disease [139,140]. Platelets are a major repository of P-selectin, an adhesion molecule expressed on the platelet surface [141,142,143]. Platelet-malignant cell adhesion is mediated i.a. by P-selectin [144,145,146]. Figure 3 shows the P-selectin centered amplification loop where platelets’ P-selectin mediates accretion of tumor trophic platelets, monocytes, and neutrophils that in turn increase platelet delivery of P-selectin.

P-selectin is also expressed on vessel endothelial cells, where it mediates platelet and neutrophil adhesion. Interestingly, cilostazol decreased P-selectin expression on endothelial cells [147]. Cilostazol also reduces adverse cardiovascular events in humans by effecting the vessel wall endothelium and triglyceride reduction, seemingly independent of any platelet effects [148,149,150,151].

The second-most remarkable finding vis a vis platelets, cilostazol, and cancer was an in vitro study by Suzuki et al. They showed that cilostazol inhibited in vitro invasion of pancreas cancer cells by reducing those cells’ synthesis of matrix metalloproteinase-9 [152]. Their conclusion was to “…propose that antiplatelet agents are applicable in clinical treatment to inhibit metastasis of malignant tumor cells”.

Perhaps the most remarkable finding regarding platelets in cancer physiology is that platelets inject their own mitochondria into cancer cells, demonstrated in osteosarcoma and breast cancer but probably do so universally throughout the common cancers [153,154,155,156]. Such transfer was platelet-to-cancer cell adherence dependent, and adherence was mediated in part by P-selectin. Although cilostazol inhibits P-selectin release, it is unknown if that is sufficient to limit platelet mitochondrial transfer to a cancer. If such bird-like feeding of cancer cells occurs throughout the common cancers, this would be a finding of the first magnitude with fundamental treatment consequences. Platelet transfer of viable, respiratory competent mitochondria also occurs in normal wound healing, forming, in part, the basis for platelet facilitation of wound healing [157,158,159].

Caveat: Since (i) aspirin, (ii) cilostazol, and (iii) the clopidogrel-group of antiplatelet drugs all reduce platelet aggregation and mediate platelets’ contribution to thrombosis by their respective three different mechanisms, we cannot yet assume that data reviewed here for cilostazol would apply to other thrombosis inhibiting medicines in current clinical use.

The reviewed data in this platelet section imply two different effects of a growing cancer on platelets:

(i) increased absolute platelet count and elevated PLR imply communication of a cancer with bone marrow, and

(ii) activation and attraction of platelets to a growing cancer imply a cancer cell mediated change in platelet function.

## 7. Discussion

Many common cancers have ALK overdrive as one part of their suite of growth driving elements, thus making pharmacological or other ALK inhibition a potential “tumor agnostic target” [160,161]. This implies potential usefulness of adding an adjunctive IC Regimen to lorlatinib treatment of other ALK positive cancers like glioblastoma or neuroblastoma.

Aspirin inhibits platelet aggregation and platelet activation as a consequence of its COX-1 inhibition. Cilostazol inhibits platelet activation by PDE 3 inhibition. Interference with platelet function also differs between aspirin and cilostazol, as evidenced by the greater bleeding risk with aspirin use compared to cilostazol.

We do not know yet to what degree, or even if, cilostazol will limit platelet delivery of growth and metastasis stimuli to a growing cancer in clinical practice. Regarding concerns of the absence of any clinical trials of cilostazol added to cancer treatment, we can balance that absence with:

(i) the few papers that do show cancer growth retarding effect of cilostazol in animal models [96,152,162,163,164,165,166,167,168]

(ii) the strength of rationale for its use.

(iii) the demonstrated low risk of bleeding or other adverse events.

In terms of the Second Preface to this paper, lorlatinib would be the decisive operation, and itraconazole and cilostazol the shaping operations.

As in Figure 1, Hh has two amplification feedback loops that would potentially increase strength of ALK expression and signaling, making Hh inhibition a particularly attractive physiological point to deepen inhibition of ALK alongside lorlatinib.

Since shifting dependence on ALK to alternate or parallel signaling forms one of the resistance pathways to lorlatinib, and platelets comprise a trophic source of many of these parallel signaling agonists, cilostazol has potential to delay lorlatinib resistance.

## 8. Conclusions

Three amplification feedback loops in ALK signaling

(i) MET→ALK→MET→ALK→…

(ii) ALK→Hh→ALK→Hh→…

(iii) Hh→Gli-A→Hh→Gli-A→…

conspire to maintain pathological ALK growth drive. Repurposed itraconazole and cilostazol added to lorlatinib may retard development of lorlatinib resistance. The potential of itraconazole increasing lorlatinib levels and thereby creating adverse events merits caution and close monitoring.

## Figures and Tables

**Table 1:** General medical use and use during lorlatinib treatment in NSCLC/LCNEC.

Drug	General Medical Use	Use with Lorlatinib
itraconazole	anti-fungal	Hh, p-gp, 3A4 inhibition
cilostazol	thrombosis prevention	growth factor deprivation

Hh, Hedgehog signaling complex.

**Table 2:** Basic pharmacological parameters of the IC Regimen drugs.

Drug	T1/2	Metabolism	Inhibition of	Side Effects
itraconazole	1 d	3A4	3A4, p-gp	⇧LFT
cilostazol	12 h	3A4, 2C19	none	headache, diarrhea
lorlatinib	1 d	3A4, UGT1A4	none	hyperlipidemia neuro-psyche

LFT = increased liver transaminases; T1/2 times are approximate and vary from individual to individual.

## Abbreviations

ALK, anaplastic lymphoma kinase; Hh, Hedgehog signaling complex; IGF, insulin-like growth factor; LCNEC, large cell neuroendocrine lung cancer; NSCLC, non-small cell lung adenocarcinomas; PDE3, phosphodiesterase 3
